# Heat Stress Tolerance Gene *FpHsp104* Affects Conidiation and Pathogenicity of *Fusarium pseudograminearum*

**DOI:** 10.3389/fmicb.2021.695535

**Published:** 2021-07-28

**Authors:** Huiqing Xia, Linlin Chen, Zhuo Fan, Mengya Peng, Jingya Zhao, Wenbo Chen, Haiyang Li, Yan Shi, Shengli Ding, Honglian Li

**Affiliations:** ^1^College of Plant Protection, Henan Agricultural University, Zhengzhou, China; ^2^National Key Laboratory of Wheat and Maize Crop Science, Zhengzhou, China

**Keywords:** *Fusarium* crown rot, *Fusarium pseudograminearum*, heat tolerance, Hsp104, pathogenesis

## Abstract

Heat shock protein Hsp104, a homolog of the bacterial chaperone ClpB and plant Hsp100, plays an essential part in the response to heat and various chemical agents in *Saccharomyces cerevisiae*. However, their functions remain largely unknown in plant fungal pathogens. Here, we report the identification and functional characterization of a plausible ortholog of yeast Hsp104 in *Fusarium pseudograminearum*, which we termed FpHsp104. Deletion mutant of FpHsp104 displayed severe defects in the resistance of heat shock during *F. pseudograminearum* mycelia and conidia when exposed to extreme heat. We also found that the protein showed dynamic localization to small particles under high temperature. However, no significant differences were detected in osmotic, oxidative, or cell wall stress responses between the wild-type and Δ*fphsp104* strains. Quantitative real-time PCR analysis showed that *FpHsp104* was upregulated in the conidia, and disruption of *FpHsp104* gene resulted in defects in conidia production, morphology, and germination. The transcript levels of conidiation-related genes of *FpFluG*, *FpVosA*, *FpWetA*, and *FpAbaA* were reduced in the Δ*fphsp104* mutant vs. the wild-type strain, but heat-shocked mRNA splicing repair was not affected in Δ*fphsp104*. Moreover, Δ*fphsp104* mutant also showed attenuated virulence, but its DON synthesis was normal. These data from the first study of Hsp104 in *F. pseudograminearum* strongly suggest that *FpHsp104* gene is an important element in the heat tolerance, development, and pathogenicity processes of *F. pseudograminearum*.

## Introduction

*Fusarium pseudograminearum* is a soil-borne plant pathogen that causes *Fusarium* crown rot (FCR) in wheat and barley. FCR has caused serious economic losses worldwide (Kazan and Gardiner, 2018), particularly in the Huanghuai wheat-growing region of China, and is considered to be one of the most destructive diseases of wheat (Li et al., 2012; Zhou et al., 2019). *F. pseudograminearum* is a hemibiotrophic pathogen, and its most common route of infection of wheat is initiated at the coleoptile by mycelia and spores in soil. Then, the pathogen moves into the hypodermis to induce typical browning of the stem, after which it can move from the stem base to the heads through the pith parenchyma (Knight and Sutherland, 2013, 2016). Similar to other fusaria, *F. pseudograminearum* can produce numerous bioactive secondary metabolites (Blum et al., 2019; Wollenberg et al., 2019; Kang et al., 2020). For instance, the type-B trichothecene DON (3-acetyl and 15-acetyl-deoxynivalenol) is an important metabolite produced during infection (Tunali et al., 2012; Obanor and Chakraborty, 2014). Moreover, *F. pseudograminearum* can cause *Fusarium* head blight in wheat, especially under warm and humid conditions (Obanor et al., 2013).

Thermal tolerance is a basic determinant of an organism’s ecology, and temperature can affect a species’ abundance, spatiotemporal distribution, habitat colonization, and interactions (Robert et al., 2015; Scafaro et al., 2016; Noer et al., 2020; Padfield et al., 2020; Geange et al., 2021). In response to high-temperature stress, eukaryotes trigger the expression of a number of networking genes, those encoding including heat shock proteins (Hsps) (Tsiomenko and Tuimetova, 1995; Leuenberger et al., 2017; Cha et al., 2020; Ding et al., 2020). Hsps are classified according to their approximate molecular masses, for instance, Hsp100, Hsp90, Hsp70, Hsp60, and small Hsps (Ohtsuka et al., 2007). Hsp104 is a member of the Clp/Hsp100 family, which includes ClpB in bacteria and Hsp100 in plants (Sanchez and Lindquist, 1990; Hodson et al., 2012; Mishra and Grover, 2016). Like other Clp/Hsp100 chaperones, Hsp104 is a hexameric AAA^+^ ATPase that couples ATP hydrolysis to drive energy-expensive processes such as DNA unwinding, mRNA splicing, and chromatin condensation, as well as protein transport, disaggregation, and degradation (Vogel et al., 1995; Shorter, 2008; Sweeny et al., 2020).

In *Saccharomyces cerevisiae*, Hsp104 responds to heat, ethanol exposure, and other stresses (Kempf et al., 2017). At normal temperatures, Hsp104 is present in small amounts in the cytoplasm and nucleus, and Hsp104 formed aggregates with increasing time on exposure to mild heat shock. However, Hsp104 is not expressed in lethally heat-shocked cells (Howie et al., 2019). In response to heat shock, the N-terminal region of Hsp104 destabilizes the yeast prions [PSI+] in a Sir2-dependent process by artificial overproduction of Hsp104 (Howie et al., 2019; Kryndushkin et al., 2011; Rosenzweig et al., 2015). Furthermore, Hsp104 shows plasticity in disaggregating diverse substrates; these are enveloped inside the axial channel of Hsp104, which forms dynamic hexamers that adopt open “lock-washer” spiral states (Shorter and Southworth, 2019). In fungal pathogen *Candida albicans*, no evident growth or morphological defects resulted from locking Hsp104, but biofilm formation showed structural defects in the Δ*hsp104* mutant, the virulence of which was decreased in a worm infection model (Fiori et al., 2012).

Pre-mRNA splicing is also disrupted by severe heat shock in *S. cerevisiae* and other eukaryotic cells (Biamonti and Caceres, 2009). Hsps have the characteristic attribute of having their expression modified by or alternatively protecting pre-mRNA splicing. For example, Hsp70 binds to U-rich RNA elements to stabilize certain transcripts in cells, independent of its protein chaperone cycle (Kishor et al., 2013; Yamamoto et al., 2016). Hsp60s are involved in the RNA splicing of rpl2 and ccmFC introns in mitochondria via their RNA-binding ability (Hsu et al., 2019). Hspl04 promotes the recovery of heat-damaged splicing, with much more rapid recovery observed in wild-type (WT) strains compared with strains containing *hsp104* mutations when splicing in *S. cerevisiae* was disrupted by heat shock. Moreover, Hsp105 (also known as Hsph1) pre-mRNAs are alternatively spliced in response to heat stress in mammalian cells (Takechi et al., 1994). However, the functions of Hsp104 in plant fungal pathogens have remained generally unknown.

In the present study, we identified and functionally characterized a putative *Hsp104* gene in *F. pseudograminearum*, named *FpHsp104*. The *FpHsp104*-deletion mutant exhibited reduced heat resistance and decreased conidiation, and its conidial morphology was abnormal. The Δ*fphsp104* mutant showed attenuated virulence toward wheat and barley plants, with reduced conidia germination and infection hyphal development. Overall, FpHsp104 was important for heat stress tolerance, conidiation, and pathogenicity in *F. pseudograminearum*.

## Materials and Methods

### Strains and Culture Conditions

The *F. pseudograminearum* Wz2-8A strain was used as the WT in this study (Zhou et al., 2019). All strains were maintained on potato dextrose agar (PDA) medium at 25°C for standard culturing. Liquid YEPD (1% tryptone, 0.3% yeast extract, and 2% glucose) medium was used to collect fungal mycelia for genomic DNA and RNA extraction. TB3 (3 g of yeast extract, 3 g of casamino acids, 200 g of sucrose, and 10 or 15 g of agar in 1 L of distilled water) medium with 100 μg/ml of hygromycin B (Roche, Basel, Switzerland) or 100 μg/ml of G418 (Invitrogen, Carlsbad, CA, United States) was used for protoplast regeneration. CMC (15 g of carboxymethyl cellulose, 1 g of NH_4_NO_3_, 1 g of KH_2_PO_4_, 1 g of yeast extract, and 0.5 g of MgSO_4_⋅7 H_2_O in 1 L of distilled water) medium was used to induce conidia at 25°C and 150 rpm for 4 days.

### Sequence and Phylogenetic Analyses of *FpHsp104* From *Fusarium pseudograminearum*

The Hsp104 (NP_013074.1) protein from *S. cerevisiae* and Hsp98 (XP_957228.1) protein from *Neurospora crassa* (Vassilev et al., 1992) were used as the query to search the *F. pseudograminearum* genome (Gardiner et al., 2018) by BlastP algorithms. The ortholog *FpHsp104* (*FPSE_03525*) was blasted and downloaded from National Center for Biotechnology Information (NCBI). FpHsp104 homologs from *Chaetomium thermophilum* (XP_006692738.1), *Magnaporthe oryzae* (XP_003717107.1), and *Fusarium graminearum* (XP_011324022.1) identified in NCBI were downloaded and used to construct neighbor-joining trees for Hsp104 with the MEGA 5.05 software package, using the neighbor-joining method with 1,000 replicates for bootstrap analysis. ClpB from *Escherichia coli* and Hsp101 from *Arabidopsis thaliana* were used as the out-group. The domains of these proteins were predicted using the SMART website.^1^

### Quantitative Real-Time PCR Analysis

To analyze *FpHsp104* expression during infection, WT conidia were collected in CMC liquid. Wheat coleoptiles were inoculated with 1 × 10^7^ conidia/ml conidial suspensions for 18, 30 h, 2, 3, 5, and 7 days. To test *FpHsp104* expression under heat shock pressure, the WT conidia were first cultured in YEPD at 25°C for 12 h, heated to 34°C for 1 h, and then returned to 25°C for 0, 3, 6, 12, 24, or 48 h. To determine the expression of conidiation-related genes in the WT, Δ*fphsp104*, and Δ*fphsp104*-C strains, conidia were produced in CMC at 25°C for 3 days. Total RNA from lyophilized mycelia, conidia, and infected plants was extracted using a KKFast plant RNApure Kit (Zomanbio, Beijing, China) and transcribed into complementary DNA (cDNA) with HiScript III RT SuperMix for qPCR (+ gDNA wiper) (Vazyme, Nanjing, China). Each cDNA was used as a template for qRT-PCR analysis using ChamQ Universal SYBR qPCR Master Mix (Vazyme, Nanjing, China). *FpTEF1a* and *FpActin* genes were used as internal standards. The relative normalized transcript level of each gene was computed using the 2^–ΔΔCt^ method. The primers used for qRT-PCR are listed in Supplementary Table 1. Data were analyzed using *t*-tests in Excel.

### Construction of the Δ*fphsp104* Mutant and Complementary Strains

**FIGURE 1:**
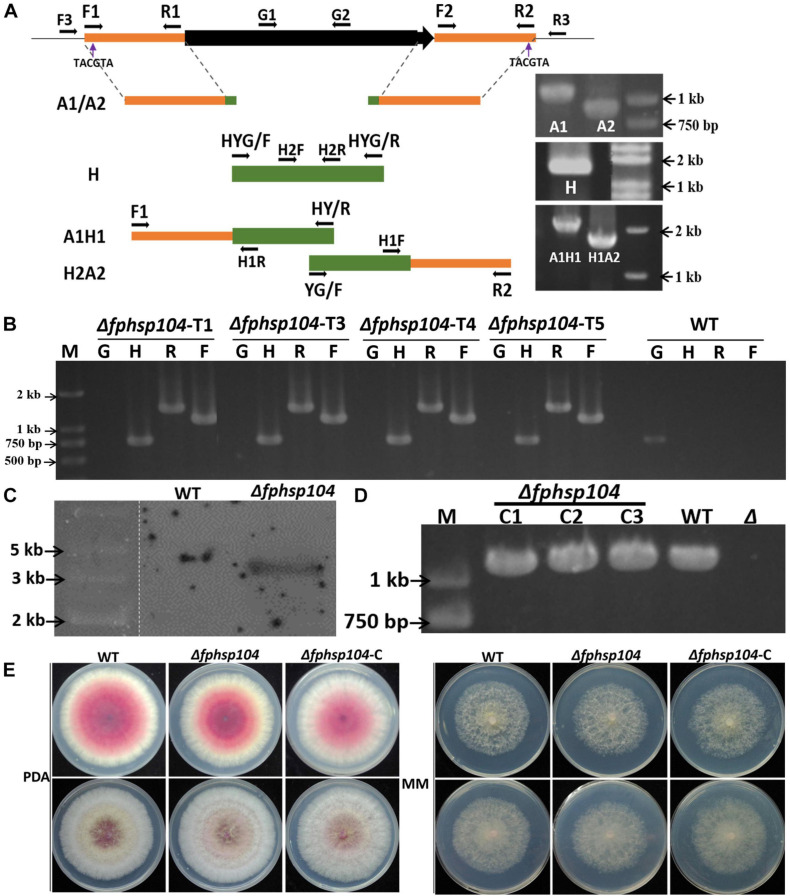
Targeted gene replacement of *FpHsp104* and genetic complementation. **(A)** Scheme for Δ*fphsp104* strain construction by homologous recombination. The 903-bp 5′-UTR (A1) and 1,108-bp 3′-UTR (A2) of *FpHsp104* from *Fusarium pseudograminearum* and 1,380 bp *hph* (H) were amplified, respectively. Two fusion products, A1H1 (1,378 bp) and A2H2 (1,751 bp), were amplified by overlapping PCR. **(B)** Results of PCR analysis, in which a 750-bp fragment of the *hph* gene (H), a 1,118-bp fragment of *FpHsp104* and *hph* upstream cassette (F), and a 1,436-bp fragment of *FpHsp104* and *hph* downstream cassette (R) were amplified from Δ*fphsp104* mutants. A 1,087-bp fragment of the *FpHsp104* gene was amplified from the wild-type (WT) strain. **(C)** Southern blotting analysis of WT and Δ*fphsp104*-T4 strains with a 750-bp fragment as a probe. The genomic DNA of each strain was digested with *Sna*BI. **(D)** Complementation strains were detected with PCR analysis, in which a 1,087-bp fragment of the *FpHsp104* gene was amplified. **(E)** Phenotypes of WT, Δ*fphsp104*, and Δ*fphsp104*-C strains. All strains were inoculated on potato dextrose agar (PDA) and minimal medium (MM) for 3 days at 25°C.

The split-PCR approach (Catlett et al., 2003) was used to generate *FpHsp104* gene-replacement constructs (Figure 1A). Flanking sequences of 903 base pairs (bp) upstream and 1,108 bp downstream (A1/A2) of *FpHsp104* gene were amplified by PCR with primer pairs F1 + R1 and F2 + R2 (Supplementary Table 1), respectively. The hygromycin phosphotransferase (*hph*) gene (H) was amplified by PCR with primer pair HYG/F + HYG/R. These three fragments were used as templates for overlapping PCR amplification to obtain a 1,378-bp fragment containing the *FpHsp104* and *hph* upstream cassette (A1H1) and a 1,751-bp fragment containing the *FpHsp104* and *hph* downstream cassette (H2A2), respectively. The resulting PCR products were purified and transformed into protoplasts by the polyethylene-glycol-mediated fungal transformation method (Liu and Friesen, 2012). *F. pseudograminearum* protoplasting and transformantions were performed previously described (Wang et al., 2017).

The transformants were screened on PDA medium supplemented with hygromycin B (100 μg/ml). The putative transformants were confirmed by PCRs with the primer pairs H2F + H2R, F3 + H1R, H1F + R3, and G1 + G2 (Supplementary Table 1) and Southern blotting. Southern blotting was performed using a DIG High Prime DNA Labeling and Detection Starter kit I (Roche Diagnostics, Mannheim, Germany) according to the manufacturer’s instructions. A 750-bp fragment of *hph* gene amplified with primer pair H2F + H2R (Supplementary Table 1) was used to prepare the probes and detect the genomic DNA digested with *Sna*BI.

The complementation assay was conducted with *FpHsp104* gene driven by 1,087-bp native promoter amplified by primer pair G1 + G2 (Supplementary Table 1) and fused with *GFP* in C terminal in pKNTG vector (Ye et al., 2014) following ClonExpress II one-step cloning kit C112 (Vazyme, Nanjing, China). The resulting complementation construct was introduced into mutant protoplasts using the same approach as above. The transformants with G418 resistance were further confirmed by PCR with the primer pair neiF + GFPR (Supplementary Table 1) and fluorescence detection by microscopy (Nikon Eclipse Ti-S; Nikon, Tokyo, Japan). Fluorescent conidiophores, conidia, and germinated conidia expressing FpHsp104-GFP were examined under optimal temperature (25°C) or high temperature (34°C) for 20 min with the aid of a CARL ZEISS (Oberkochen, Germany) Axio imager M2 microscope. DAPI (Invitrogen, United States) was used for nuclear staining, and FM4-64 (Invitrogen, United States) was used for vacuole staining.

### Vegetative Growth and Stress Response Assays

Vegetative growth of the WT, Δ*fphsp104*, and Δ*fphsp104*-C strains was measured on PDA and minimal medium (MM) agar plates. Mycelial plugs (5 mm × 5 mm) from 3-day-old PDA/MM plates were transferred onto new plates at 25°C. Colonies were photographed and measured at 3 days. For heat response analysis, mycelial plugs of equal size were inoculated on PDA/MM plates at 25°C for 36 h, at 34°C for 24 h, and then at 25°C again for 2 days. Mycelial morphology was observed using a CARL ZEISS Axio imager M2 microscope. For stress response assays, mycelial plugs of equal size were placed on freshly prepared PDA plates with 1 mM of CuSO_4_, 10 mM of MnCl_2_, 0.05% sodium dodecyl sulfate, and 5 mM of hydrogen peroxide or 50 mg/ml of congo red for 3 days at 25°C in the dark. All experiments were repeated three times, with three replicates each time. Data were analyzed using *t*-tests in Excel.

### Conidiation and Germination Assays

For conidiation, the WT, Δ*fphsp104*, and Δ*fphsp104*-C strains were inoculated on PDA plates at 25°C for 2 days; then, 5-mm-diameter mycelial plugs were punched from the edges of plate colonies and transferred into liquid CMC. Mycelia were cultured with shaking (150 rpm) at 25°C for 4 days. Conidiophore morphology was observed using a CARL ZEISS Axio imager M2 microscope. Conidia were filtered through one layer of miracloth and counted with a hemacytometer under a CARL ZEISS Axio imager M2 microscope. Calcofluor White stain (MERCK, Darmstadt, Germany) was used for septa staining. Conidia were shocked in 34°C condition for 3 h and assayed using propidium iodide (PI) (Solarbio, Beijing, China). Stained cells were imaged and counted using a CARL ZEISS Axio imager M2 microscope. PI-positive cell proportions were determined after examining at least 500 cells in each of three independent experiments. Conidia were shocked in 34°C condition for 2 h and inoculated on MM plates at 25°C for 3 days to measure the mycelia growth.

Conidial suspensions of 100 μl (5 × 10^4^ conidia/ml) were, respectively, prepared by distilled water (ddH_2_O), potato dextrose broth (PDB) media, or MM media and dropped onto the surface of a slide, which was placed in a moistened box at 25°C. The conidial germination rate was determined as percentage of germinated conidia to total conidia. More than 200 germinated conidia were counted for each strain, and slides were photographed at 3–6 h under the microscope; more than 200 germinated conidia were counted for each strain in each of three independent experiments. Data were analyzed using *t*-tests in Excel.

### mRNA Splicing Analysis

To analyze mRNA splicing in the WT, Δ*fphsp104*, and Δ*fphsp104*-C strains, conidia were cultured in YEPD at 25°C for 12 h, at 34°C for 2 h or not, and then at 25°C again for 0, 1, or 2 h. Northern blotting hybridization was used to analyze the mRNA of the *F. pseudograminearum* actin gene (*FPSE_04141*) with a DIG Northern Starter kit (Roche, Switzerland).

### Pathogenicity Assays

Seedlings of the wheat cultivar Aikang 58 were grown at 25°C with a photoperiod of 16 h/8 h (light/dark cycle) for 4 days. Barley was cultured under the same conditions for 7 days. Then, 5-mm-diameter mycelial plugs were punched from the edges of plate colonies and inoculated on wheat coleoptiles and barley leaves. Agar plugs were used for negative control. The mycelial plugs were removed after 24 h post-inoculation, and lesion formation was examined at 4 days post-inoculation (dpi). Inoculated wheat epidermal cells were viewed under a Nikon Ti-s microscope. Post-cultural infection assays were carried out as previously described (Chen et al., 2019). All experiments were repeated three times, with three replicates each time. Data were analyzed using *t*-tests in Excel.

### DON Enzyme-Linked Immunosorbent Assay

The WT, Δ*fphsp104*, and Δ*fphsp104*-C strains were inoculated on PDA plates at 25°C for 3 days; millet seed was sterilized at 121°C for 20 min and then inoculated with five 5-mm-diameter *F. pseudograminearum* mycelial plugs at 25°C for 7 days. Flasks were shaken daily until the mycelia colonized the millet. In controls, sterile millet was used. DON contamination in millet was assayed using an ELISA kit from Huaan Magnech Bio-Tech, Beijing, China, strictly following the manufacturer’s instructions. The experiment was repeated three times for each strain, and data were analyzed using *t*-tests in Excel.

## Results

### Identification and Expression of *FpHsp104*

**FIGURE 2:**
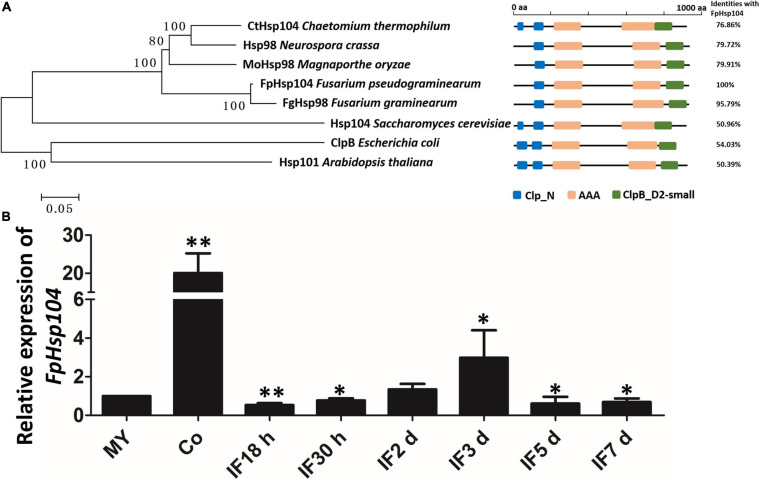
Phylogenetic relationships, domain organization, and gene expression analysis of FpHsp104 in *Fusarium pseudograminearum*. **(A)** Phylogenetic tree and domain analyses of selected known Hsp104 proteins. The neighbor-joining tree of fungal Hsp104 orthologs was constructed using the MEGA 5.05 program. Domain annotation was performed using the SMART website. **(B)** Transcription of *FpHdp104* in the infected host was assayed using qRT-PCR. MY, mycelia; Co, conidia; IF 18 h to 7 d, the time points (hours or days) post-infection. Expression levels represent *FpHsp104* mRNA levels relative to those of *FpTEF1a* gene. Bars represent standard deviation based on four replicates.^∗∗^*p* < 0.01, ^∗^*p* < 0.05 (*t*-test).

Previous studies have suggested that Hsps are conserved from yeasts to animals and plants. Therefore, we searched available genomes of *F. pseudograminearum* (Gardiner et al., 2018) and identified *FPSE_03525* as a homolog of *S. cerevisiae Hsp104* and *N. crassa Hsp98*, which we named *FpHsp104*. FpHsp104 consists of 924 amino acids with a predicted molecular mass of 103.07 kDa and isoelectric point of 5.64. A phylogenetic tree of FpHsp104 and its orthologs in five other fungi was constructed using MEGA 5.05 (Figure 2A). The Hsp104 from *F. graminearum* and that of *F. pseudograminearum* had the closest genetic relationship, and the Hsp104 protein sequences from filamentous fungi were classified into a separate cluster from those from the unicellular fungal *S. cerevisiae*. The protein domain in Hsp104 was further analyzed using the SMART website; two AAA domains were found across all species (Figure 2A), indicating that Hsp104 might have conserved roles.

The expression levels of *FpHsp104* were monitored by qRT-PCR during vegetative growth, conidiation, and the plant-pathogenic process (Figure 2B and Supplementary Figure 1). When normalized by the expression level in the vegetative mycelium stages (*FpTEF1a* as the internal standard), transcripts of *FpHsp104* showed a 20-fold increase in conidia (Figure 2B). Expression levels of *FpHsp104* were stable in almost all infection stages, yet there was a threefold increase at 3 dpi (Figure 2B). Furthermore, the relative expression levels of *FpHsp104* gene were validated using *FpActin* gene as the internal standard by qRT-PCR (Supplementary Figure 1). The transcriptional levels of *FpHsp104* escalated in conidia and 3 dpi, which are comparable between the two qRT-PCR assays. Such patterns of gene expression suggest differential roles of *FpHsp104* in *F. pseudograminearum* conidiation and pathogenesis.

### Deletion of *FpHsp104* Does Not Affect the Hyphal Growth

To determine the functions of FpHsp104 in *F. pseudograminearum*, *FpHsp104*-deletion strains (Δ*fphsp104*) were constructed by transforming WT protoplasts with the fusion PCR products (Figure 1A). The resulting transformants were confirmed by PCR analysis (Figure 1B). The PCR results showed that *FpHsp104* had been deleted from the Δ*fphsp104* mutants, and amplification of *hph* gene and the flanking regions between *hph* and the 5′ untranslated region (5′-UTR) and 3′-UTR of *FpHsp104* indicated that *FpHsp104* had been replaced by *hph* gene (Figure 1B). The Δ*fphsp104* mutant T4 was further confirmed by the single insertion by Southern blotting analysis (Figure 1C), and no *FpHsp104* expression was determined by qRT-PCR (Supplementary Figure 2). Thus, T4 was selected for FpHsp104 complementation. Complementary strains (Δ*fphsp104*-C), containing the full-length sequence except the stop codon of FpHsp104 and GFP under the control of the FpHsp104 native promoter, were also generated (Figure 1D).

To determine whether the function of FpHsp104 is associated with growth and morphogenesis in *F. pseudograminearum*, we investigated growth rates and colony morphology on PDA and MM media. As shown in Figure 1E, the hyphal growth rate and colony morphology of Δ*fphsp104* were similar to those of the WT and complementary strains. The results suggest that FpHsp104 is dispensable for hyphal growth.

### *FpHsp104* Is Critical for Heat Shock Tolerance

**FIGURE 3:**
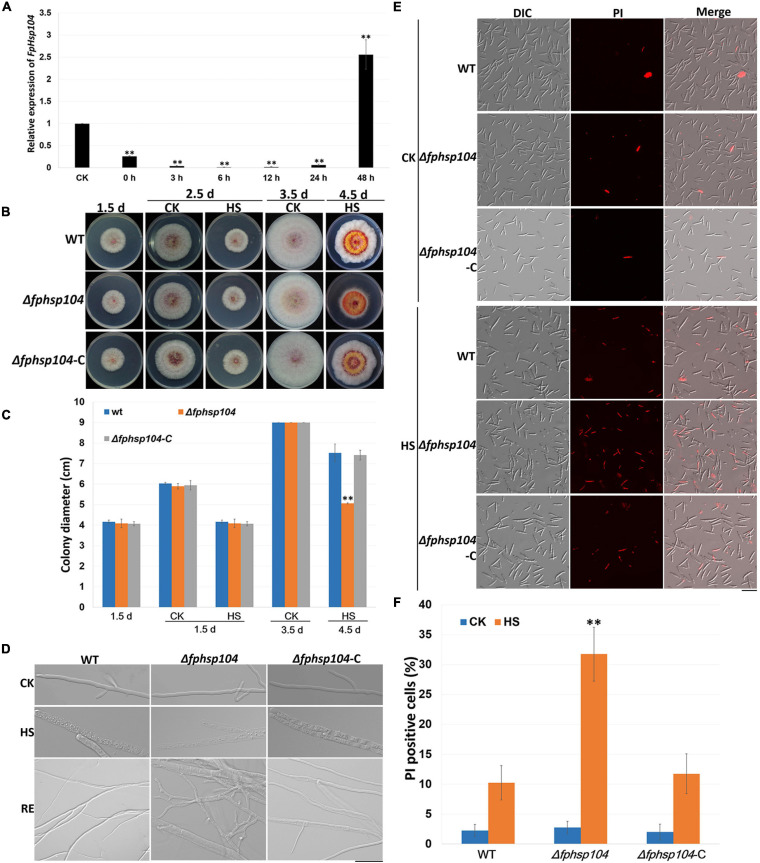
Heat-shock-tolerance assays. **(A)** Relative expression levels of *FpHsp104* in *Fusarium pseudograminearum* wild-type (WT) strain cultured in YEPD at 25°C for 12 h (CK), exposed to 34°C for 1 h, and returned to the optimal temperature (25°C) for 0–48 h. Expression levels represent *FpHsp104* mRNA levels relative to those of *FpTEF1a* gene. Bars represent standard deviation based on four replicates. ^∗∗^*p* < 0.01, ^∗^*p* < 0.05 (*t*-test). **(B)** Phenotypes of WT, Δ*fphsp104*, and Δ*fphsp104*-C strains cultured on potato dextrose agar (PDA) at 25°C for 1.5 days, 2.5 days (CK), and 3.5 days (CK); exposed to 34°C for 1 day (2.5 d-HS); and returned to the optimal temperature (25°C) for 2 days (4.5 d-HS). **(C)** Statistical analysis of colony diameter. Colony diameter is presented as the mean ± standard deviation of at least three independent experiments. ^∗∗^*p* < 0.01, ^∗^*p* < 0.05 (*t*-test). **(D)** Hyphal morphology of WT, Δ*fphsp104*, and Δ*fphsp104*-C strains cultured on PDA at 25°C (CK), exposed to 34°C for 1 day (HS), and returned to 25°C for 2 days (RE). Bar = 20 μm. **(E)** Propidium iodide (PI) staining of WT, Δ*fphsp104*, and Δ*fphsp104*-C conidia exposed to high temperature (34°C) (HS) or not (CK). PI-positive cells showed red fluorescence. **(F)** PI-positive cell numbers of WT, Δ*fphsp104*, and Δ*fphsp104*-C strains, exposed to 34°C (HS) or not (CK). Bars show mean values with standard deviation. ^∗∗^ Significant difference at *p* < 0.01 using *t*-test.

It is known that Hsp104 plays a crucial role in the survival of cells exposed to high temperatures and other severe stresses in yeast (Grimminger-Marquardt and Lashuel, 2010). To investigate the roles of FpHsp104 in heat shock tolerance, qRT-PCR was used to determine the transcript levels of *FpHSP104* in *F. pseudograminearum* incubating under high-temperature conditions. The WT conidia were germinated in YEPD for 12 h, exposed to 34°C for 1 h, and then returned to the optimal temperature (25°C). Expression of FpHsp104 was inhibited by exposure to the lethal temperature and then substantially upregulated after the temperature was returned to 25°C for 36–48 h (Figure 3A), suggesting that FpHsp104 may play roles in the survival of cells exposed to high temperature.

To determine whether the disruption of FpHsp104 affected the heat sensitivity of *F. pseudograminearum*, fungal tolerance to heat shock was examined. WT, Δ*fphsp104*, and Δ*fphsp104*-C mycelia were grown at 25°C and then heated to 34°C for 1 day. The growth of all strains was ceased under the lethal temperature (Figures 3B, C and Supplementary Figure 3), and microscopic observation showed that the mycelia were thicker and full of small particles, especially in the mycelia of Δ*fphsp104* (Figure 3D). However, mycelia of WT and Δ*fphsp104*-C showed recovery of growth after being returned to 25°C for 2 days, although the mycelia were thinner than those before heat shock; mycelia recovery of Δ*fphsp104* was limited and produced short curved hypha (Figures 3B–D and Supplementary Figure 3). Cell death in conidia was quantitated with the aid of the PI assay, which has been widely used to measure cell death (Crowley et al., 2016). All strains showed very few death cells under the optimum growing temperature (Figures 3E, F). However, the number of PI-positive cells of Δ*fphsp104* was significantly higher than that of WT and Δ*fphsp104*-C after the temperature was raised to 34°C for 3 h (Figures 3E, F). These results suggest that FpHsp104 is important for heat tolerance in *F. pseudograminearum*.

**FIGURE 4:**
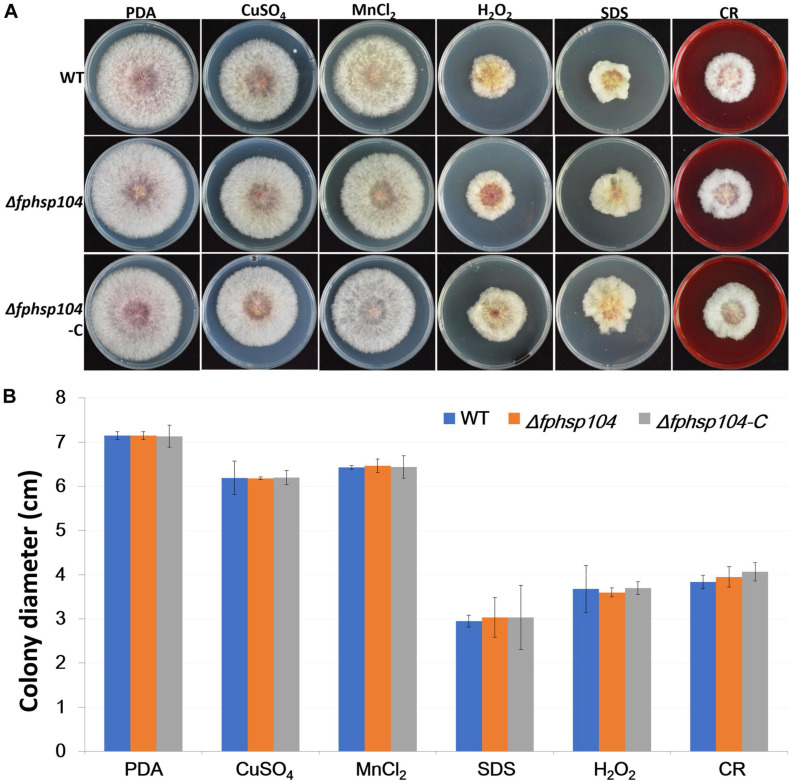
Effects of osmotic stress, oxidative stress, and cell wall stress. **(A)** Phenotypes of wild-type (WT), Δ*fphsp104*, and Δ*fphsp104*-C strains inoculated on potato dextrose agar (PDA) medium with osmotic stress factors (CuSO_4_ and MnCl_2_), oxidative stress factor (hydrogen peroxide), and cell wall stress factors (SDS and congo red [CR]). **(B)** Growth rates of the strains under osmotic, oxidative, and cell wall stresses. Results are presented as the mean ± standard deviation of at least three independent experiments. Data were analyzed using *t*-tests in Excel.

Furthermore, we assayed the growth of *F. pseudograminearum* exposed to other stresses. There were no significant differences in the growth rates of the Δ*fphsp104* mutant and the WT strain on PDA supplemented with CuSO_4_, MnCl_2_, SDS, hydrogen peroxide, and congo red (Figure 4). These results indicate that FpHsp104 is not involved in its response to a wide range of stresses.

### *FpHsp104* Shows Dynamic Localization Under High Temperature

**FIGURE 5:**
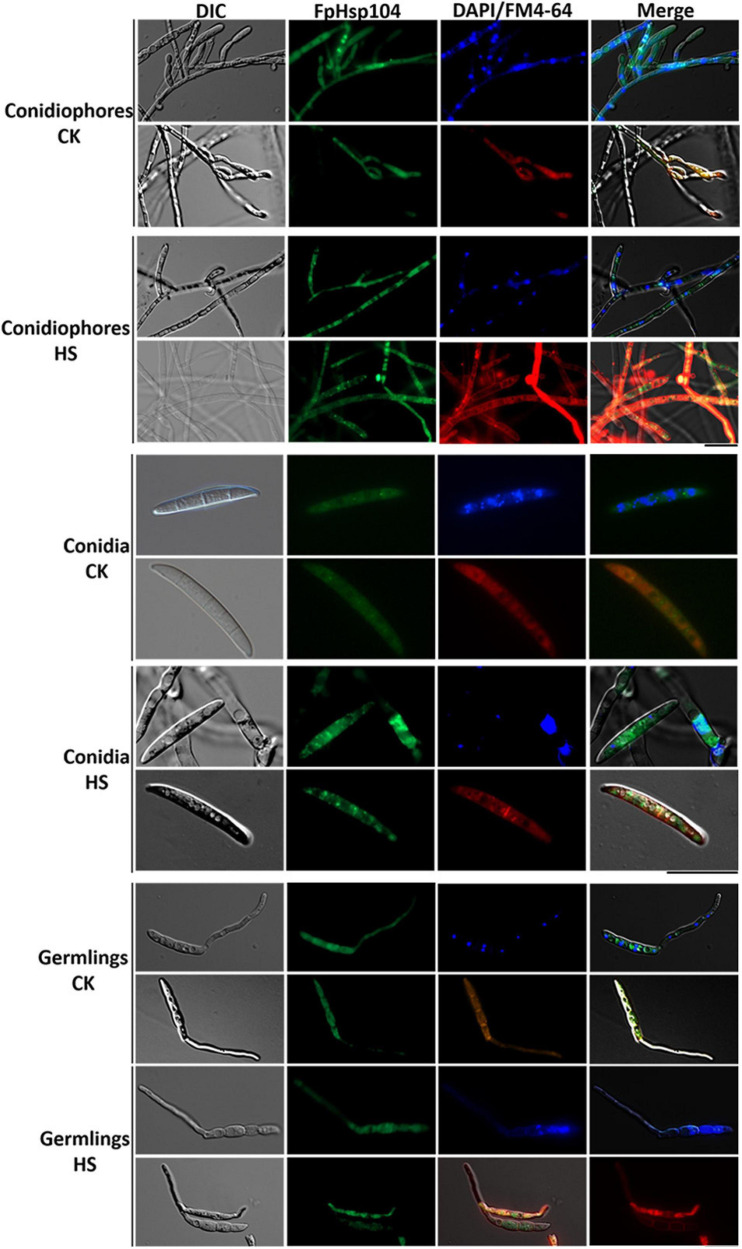
Dynamic localization of FpHsp104 under heat shock. Micrographs showing the subcellular localizations of FpHsp104-GFP exposed to 34°C (HS) or not (CK). The conidiophores (top panel), conidia (middle panel), and conidia germination (bottom panel) are shown for each treatment. DAPI and FM4-64 staining were, respectively, used to visualize nuclear and vacuole regions. Green line, GFP fluorescence intensity; blue line, DAPI fluorescence intensity; red line, FM4-64 fluorescence intensity. Bar = 20 μm.

To localize FpHsp104, FpHsp104 and its native promoter were fused with the GFP. Microscopic observation of conidiophores, conidia, and germinated conidia (germlings) of Δ*fphsp104*-C indicated that FpHsp104 was distributed throughout the cytoplasm, which also accumulated in few particles, but almost absent from the nuclei, as revealed by DAPI and FM4-64 staining (Figure 5), as is also true of *S. cerevisiae* homologs. However, high-temperature (34°C) treatment caused FpHsp104 to appear as green fluorescence punctate in conidiophores, conidia, and conidial germlings (Figure 5). The small irregular fluorescence particles may correspond to these small particles in heat-treated mycelia. FpHsp104-GFP fluorescence was not detectable in vegetative hyphae (data not shown). The results indicate that FpHsp104 might be protected from degradation under heat shock.

### *FpHsp104* Is Involved in Conidiation and Conidia Germination

**FIGURE 6:**
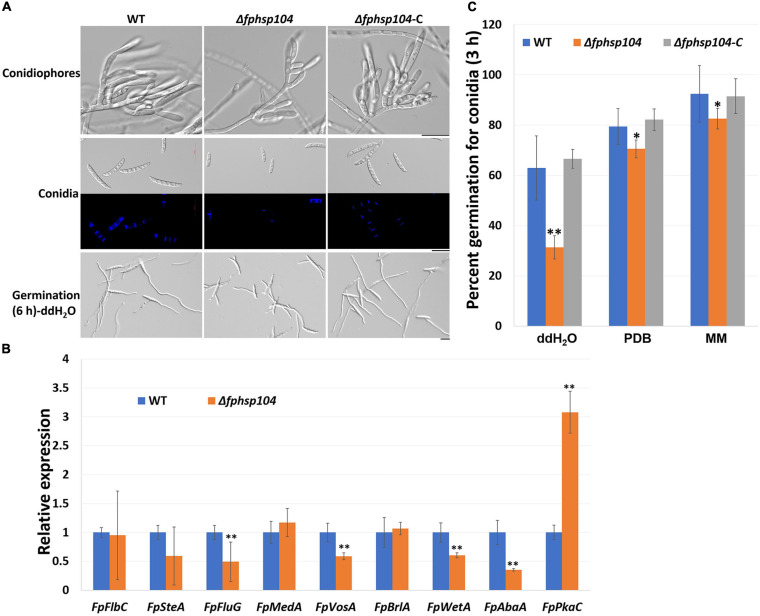
Conidiation and conidia germination assays. **(A)** Microscopy images of conidiophores and conidia produced in CMC medium for 4 days and conidia formation induced in sterile water for 6 h. Bar = 20 μm. **(B)** qRT-PCR analyses of nine conidiation-related genes in wild-type (WT) and Δ*fphsp104* strains. Transcript level fold of the selected gene was calculated relative to the level at WT. *FpTEF1a* gene was used as a reference. Bars represent standard deviation based on four replicates. ^∗∗^*p* < 0.01 (*t*-test). *FpFlbC*, FPSE_02736; *FpSteA*, FPSE_01067; *FpFluG*, FPSE_04527; *FpMedA*, FPSE_01933; *FpVosA*, FPSE_11893; *FpBrlA*, FPSE_00757; *FpWetA*, FPSE_02660; *FpAbaA*, FPSE_11664; *FpPkaC*, FPSE_00184. **(C)** Conidia germination rates of WT, Δ*fphsp104*, and Δ*fphsp104*-C strains cultured in sterile water, potato dextrose broth (PDB), and minimal medium (MM), respectively. Bars represent SD of three independent experiments. ^∗∗^*p* < 0.01, ^∗^*p* < 0.05 (*t*-test).

Conidia have an essential role in the disease cycle of *F. pseudograminearum* (Kazan and Gardiner, 2018). To assess the role of FpHsp104 in conidiation, fresh harvested hyphae of each strain were used as inoculum for liquid CMC culture at 25°C and 150 rpm. Typical conidiophores were observed in different strains, but conidia produced by Δ*fphsp104* were smaller than those of the WT and Δ*fphsp104*-C (Figure 6A). Conidial yields quantified from 4-day-old cultures of the WT, Δ*fphsp104*, and Δ*fphsp104*-C strains were 10.9 × 10^5^, 2.6 × 10^5^, and 10.3 × 10^5^ conidia/ml, respectively (Table 1), indicating a 76% decrease in conidiation capacity in the absence of *FpHsp104*. Microscopy revealed that the Δ*fphsp104* mutant also underwent some changes in conidial morphology. The lengths of the conidia from the Δ*fphsp104* mutant strain were markedly reduced, and these conidia did not display the typical slender canoe-shaped morphology of the WT (Figure 6A and Table 1). Moreover, the WT conidia usually formed 3–5 septa, whereas most of the mutant conidia had a reduced number of septa (Figure 6A and Table 1). According to the qRT-PCR results, conidiation-related genes *FpFluG*, *FpVosA*, *FpWetA*, and *FpAbaA* were downregulated in the *FpHsp104*-deletion mutant of *F. pseudograminearum* (Figure 6B and Supplementary Figure 4), indicating that *FpHsp104* might affect conidiation by regulating conidiation-related genes.

**TABLE 1:** Statistical analyses of conidia production and conidial phenotypes.

**Strains**	**Number (10^5^/ml)**	**Length (μm)**	**Septa/conidium**
WT	10.916.8	30.60.8	2.90.1
Δ*fphsp104*	2.67.5**	23.31.7**	2.20.2**
Δ*fphsp104*-C	10.312.6	29.90.7	2.960.2

WT, wild type. **p < 0.01 (t-test).

To determine whether FpHsp104 plays any part in conidial germination, we transferred freshly harvested conidia of the various strains to distilled water on slides to induce germination. Conidia germination of Δ*fphsp104* was delayed compared with that of the WT and Δ*fphsp104*-C. Approximately 65, 80, and 90% of the WT and Δ*fphsp104*-C conidia formed at 3 h in ddH_2_O, PDB, and MM, respectively, and each conidium usually formed two germ tubes from both end cells. By contrast, 31, 70, and 82% of the Δ*fphsp104* conidia produced shorter germ tubes at multiple germination sites in ddH_2_O, PDB, and MM, respectively (Figures 6A, C and Supplementary Figure 5). At 6 h, conidia of WT, Δ*fphsp104*, and Δ*fphsp104*-C formed completely in different liquids (Figure 6A and Supplementary Figure 5). Overall, these results show that FpHsp104 is important for conidiation and germ tube formation in *F. pseudograminearum*.

### No Difference in Heat-Disrupted Repair Between Wild Type and Δ*fphsp104*

**FIGURE 7:**
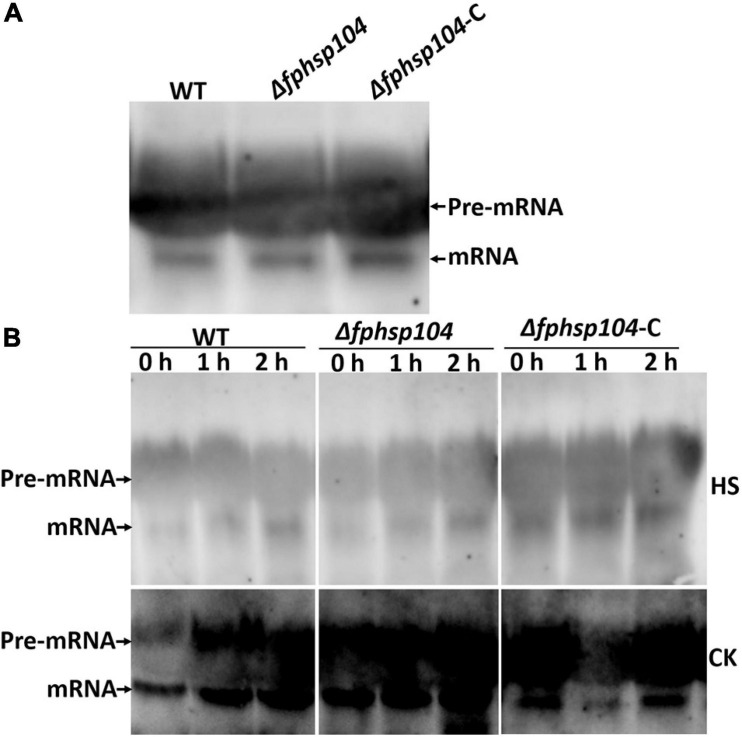
mRNA splicing after heat shock. **(A)** Conidia inoculated in YEPD at 25°C for 12 h. Total RNA of the *actin* gene was detected using Northern blotting. **(B)** Germinated conidia were heat-shocked at 34°C for 2 h (HS) or not (CK) and returned to 25°C for 0, 1, or 2 h. Total RNA of the *actin* gene was detected using Northern blotting.

Hsp104 has been reported to have roles in the repair of mRNA splicing after disruption by heat shock. The expression of some conidiation-related genes was reduced in the Δ*fphsp104* mutant. We analyzed transcripts of the stably expressing *actin* gene (*FPSE_04141*) to monitor the effects of FpHsp104 on mRNA splicing in *F. pseudograminearum*. There was no time-course change of *actin* gene mRNA splicing when *F. pseudograminearum* germinated conidia were cultured in optimal temperature, and the *actin* gene appeared similar to mRNA splicing in WT, Δ*fphsp104*, and Δ*fphsp104*-C strains (Figure 7). Conidia germlings of the WT, Δ*fphsp104*, and Δ*fphsp104*-C strains were grown at 25°C and then maintained at 34°C for 2 h. *Actin* mRNAs accumulated as unspliced precursors in both WT and mutant cells. When conidia germlings were retuned to 25°C, splicing recovered within 2 h (Figure 7). No obvious differences were detected in Δ*fphsp104* compared with the WT and Δ*fphsp104*-C strains (Figure 7), suggesting that the expression of conidiation-related genes might be regulated by FpHsp104 in other ways.

### *FpHsp104* Is Required for Pathogenicity but Not for DON Production

**FIGURE 8:**
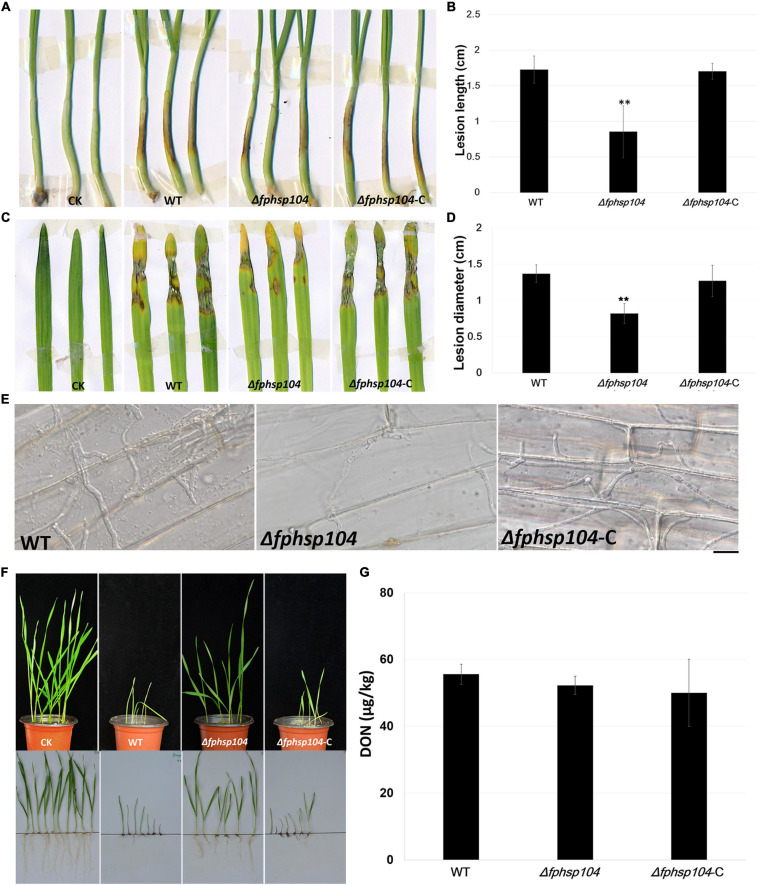
Pathogenicity and DON assays. **(A)** Wheat coleoptiles were inoculated with mycelial blocks of wild-type (WT), Δ*fphsp104*, and Δ*fphsp104*-C strains. Agar blocks without mycelia inoculated wheat as blank control (CK). **(B)** Statistical analyses of lesion lengths from the infected wheat coleoptiles. Bars represent standard deviation (SD) of three independent experiments. ^∗∗^*p* < 0.01 (*t*-test). **(C)** Barley leaves were inoculated with mycelial blocks of WT, Δ*fphsp104*, and Δ*fphsp104*-C strains. Agar blocks without mycelia inoculated barley as blank control (CK). **(D)** Statistical analyses of lesion diameters from the infected barley leaves. Bars represent SD of three independent experiments. ^∗∗^*p* < 0.01 (*t*-test). **(E)** Wheat coleoptiles from 4-day-old wheat seedlings were inoculated with mycelial blocks. Infectious growth was observed 4 days after inoculation. Bar = 20 μm. **(F)** Wheat seeds were inoculated with WT, Δ*fphsp104*, and Δ*fphsp104*-C strains and cultured at 25°C for 7 days. Sterile millet was used as blank control (CK). **(G)** DON production in WT, Δ*fphsp104*, and Δ*fphsp104*-C strains was tested using ELISA assays.

To understand the role of FpHsp104 in pathogenesis of *F. pseudograminearum*, we infected wheat coleoptiles with fresh hyphae harvested from the WT, Δ*fphsp104*, and Δ*fphsp104*-C strains. After 4 days of incubation, we recorded and quantified the disease symptoms of plants infected by each strain. The average length of brown lesions on the wheat coleoptiles infected with the Δ*fphsp104* mutant was 0.85 ± 0.36 cm, whereas those infected with the WT and Δ*fphsp104*-C strains had average lesion lengths of 1.73 ± 0.19 and 1.70 ± 0.11 cm, respectively (Figures 8A, B), indicating a significant reduction in the virulence of the Δ*fphsp104* mutant. For barley leaves, similar results were observed. The Δ*fphsp104* mutant had an average lesion diameter of 0.82 ± 0.14, compared with 1.37 ± 0.12 and 1.27 ± 0.22 cm for the WT and Δ*fphsp104*-C strains, respectively (Figures 8C, D). To further confirm the involvement of *FpHsp104* in fungal infection, penetration sites of mycelia were observed in wheat coleoptile cells at 3 dpi under a microscope. Fewer mycelia were observed in the wheat coleoptile cells inoculated with Δ*fphsp104* compared with those inoculated with the WT or Δ*fphsp104*-C strains (Figure 8E), and the penetration and extension functions of Δ*fphsp104* were inhibited. Subsequently, we inoculated wheat roots with mycelia soil of the WT, Δ*fphsp104*, and Δ*fphsp104*-C strains and obtained the same results. The virulence of the Δ*fphsp104* mutant was significantly reduced (Figure 8F). All these results indicate an important role of FpHsp104 in host infection.

Mycotoxin production is considered one of the major virulence factors in *F. pseudograminearum*. Therefore, DON production was assayed in the WT, Δ*fphsp104*, and Δ*fphsp104*-C strains. However, DON production showed no obvious changes in the Δ*fphsp104* mutant compared with the WT and Δ*fphsp104*-C strains (Figure 8G). This suggests that FpHsp104 might affect other virulence factors rather than DON production.

## Discussion

The heat-shock response is an organism’s attempt to overcome cellular stresses triggered by elevated temperatures, exposure to heavy metals, or infections; and the process is highly conserved, well ordered, and regulated. The production of a group of proteins known as Hsps can protect the cell by helping it survive under conditions that would normally be lethal. Hsp104 is highly conserved across different organisms as a heat shock-induced protein required for thermotolerance. The question whether additional features of this protein that contribute to the general fitness of the plant fungal pathogens or to its virulence may exist was raised. In this study, we identified a predicted Hsp104, named FpHsp104, which is highly conserved among organisms. *FpHsp104* was expressed at very low levels when *F. pseudograminearum* was exposed to lethal temperatures but increased when *F. pseudograminearum* was returned to the optimal temperature for 48 h, confirming previous observations on expression of Hsp104 in *S. cerevisiae*. When *FpHsp104* was disrupted, *F. pseudograminearum* showed more sensitivity to short exposures of the lethal temperature of 34°C.

At the same time, we noticed that cells were damaged and full of small particles in *F. pseudograminearum* hyphae under heat shock. Consistent with the localization of Hsp104 in other fungi, the FpHsp104-GFP fluorescence was observed in the cytoplasm of conidiophores, conidia, and germlings. Interestingly, the FpHsp104-GFP fluorescence appears in heat-treated cells as small irregular gathers. Thus, we suggest that FpHsp104 may be involved in refolding of heat-denatured proteins in the cytosol. However, Δ*fphsp104* of *F. pseudograminearum* showed a null response to osmotic stress, oxidative stress, and cell wall stress. This is in contrast to *hsp104*-deletion mutants in *S. cerevisiae*, which are involved in the response to similar chemical stress cues (Kempf et al., 2017).

Although Hsp104 is initially found that acts on a survival response to stress, recent studies have revealed its significance in biofilm formation and virulence in filamentous fungi. In *C. albicans*, Hsp104, as the heat-induced molecular disaggregase, plays roles in biofilm formation and pathogenicity (Fiori et al., 2012). However, phenotypes of *Hsp104* deletion of vary among the phytopathogenic fungi were not characterized. In *F. pseudograminearum*, the expression of *FpHsp104* was significantly upregulated in conidiation. Disruption of *FpHsp104* significantly decreased the number of conidia and resulted in marked changes in conidial morphology. Furthermore, conidiation-related genes including *FpFluG*, *FpVosA*, *FpWetA*, and *FpAbaA* were downregulated in Δ*fphsp104* mutant. In *S. cerevisiae*, Hsp104 is involved in mRNA splicing by repairing it after disruption (Yost and Lindquist, 1991; Vogel et al., 1995). However, no obvious difference in mRNA splicing was detected among the WT, Δ*fphsp104*, and Δ*fphsp104*-C strains, suggesting that FpHsp104 may regulate conidiation in other ways.

In previous studies, Hsps played a role in pathogenicity of many pathogenic fungi. In *F. pseudograminearum*, we previously reported that the endoplasmic reticulum Hsp70 protein FpLhs1 is important for plant infection and for defects in protein secretion (Chen et al., 2019). Moreover, our study confirmed that FpHsp104 also contributed to fungal virulence. DON is a well-documented virulence factor in the pathogenicity of various *Fusarium* species including *F. pseudograminearum* on wheat. However, FpHsp104 is dispensable for *F. pseudograminearum* DON synthesis. Although Hsp104 is also required for biofilm formation and pathogenicity in *C. albicans* (Fiori et al., 2012), the molecular mechanism of how the Hsp104 regulates fungal pathogenicity could not be resolved so far. Disaggregation activities of Hsp104 confer considerable selective advantages, and renaturation of aggregated conformers by Hsp104 is critical for yeast survival after various environmental stresses. As the result of FpHsp104 translocation under heat shock, we infer that structure of FpHsp104 in different conditions is likely involved in virulence of *F. pseudograminearum*.

However, many questions remain to be answered in future research. As we know, the genomes of *F. pseudograminearum* and *F. graminearum* were more than just similar, and *F. pseudograminearum* was even recognized as a population within the *F. graminearum* species group (Group 1) (Aoki and O’Donnell, 1999; Gardiner et al., 2018). Nevertheless, *F. pseudograminearum* and *F. graminearum* preferentially grow in different host tissues and conditions (Scott and Chakraborty, 2006; Kazan and Gardiner, 2018). The distribution of the two species in Huanghuai wheat-growing region of China (for example, *F. pseudograminearum* surveys conducted north of the Yangtze river) has shown that *F. pseudograminearum* is more common in cooler and drier regions, in contrast to *F. graminearum*, which is often found in warmer regions with higher rainfall (Zhou et al., 2019). We raise a question if Hsp104 contributes to their growth preference. In addition, the ATPase activity of FpHsp104, its functions in protein aggregation, and the domains responsible for certain phenotypes will be investigated in the following research.

In conclusion, we have identified a conserved Hsp104 of *F. pseudograminearum* and presented insights into its basic biological functions. The protein is indispensable for acquisition of thermotolerance and appears in heat-treated cells as small irregular gathers. The decreased proficiency of Δ*fphsp104* mutant in conidia production and formation and their attenuated virulence suggest that FpHsp104 may be a subtle regulator of cell fitness independently from its role in maintaining thermotolerance.

## Data Availability Statement

The original contributions presented in the study are publicly available. This data can be found here: (1) Hsp104 sequence from yeast was downloaded from NCBI under the accession numbers NP_013074.1. (2) Hsp104 sequences from filamentous fungi were downloaded from NCBI under the accession numbers XP_006692738.1 (Chaetomium thermophilum), XP_009254919.1 (Fusarium pseudograminearum), XP_003717107.1 (Magnaporthe oryzae), XP_011324022.1 (Fusarium graminearum), XP_957228.1 (Neurospora crassa). (3) ClpB sequence from Escherichia coli was downloaded from NCBI under the accession numbers WP_020239930.1. (4) Hsp101 sequence from Arabidopsis thaliana was downloaded from NCBI under the accession numbers NP_565083.1.

## Author Contributions

All authors listed have made a substantial, direct and intellectual contribution to the work, and approved it for publication.

## Conflict of Interest

The authors declare that the research was conducted in the absence of any commercial or financial relationships that could be construed as a potential conflict of interest.

## Publisher’s Note

All claims expressed in this article are solely those of the authors and do not necessarily represent those of their affiliated organizations, or those of the publisher, the editors and the reviewers. Any product that may be evaluated in this article, or claim that may be made by its manufacturer, is not guaranteed or endorsed by the publisher.

## Footnotes

1
http://smart.embl-heidelberg.de/
