# Supplementary material for: Heat Stress Tolerance Gene FpHsp104 Affects Conidiation and Pathogenicity of Fusarium pseudograminearum
Source: Front Microbiol. 2021 Jul 28;12:695535. doi: 10.3389/fmicb.2021.695535 (PMC8355993; doi:10.3389/fmicb.2021.695535)
Supplement: Supplementary file 1 [file Table_1.DOCX]

Table S1. Primers used in the study

| Primer | Sequence (5΄→3΄) |
| --- | --- |
| F1 | TGTCGCACCCATATCCGTA |
| R1 | TTGACCTCCACTAGCTCCAGCCAAGCCGATGGCGGTGTTGAATGGT |
| F2 | GAATAGAGTAGATGCCGACCGCGGGTACAAAAGGCAATGATTGTTA |
| R2 | CCGACTTATAATCCTCACTGC |
| HYG/F | GGCTTGGCTGGAGCTAGTGGAGGTCAA |
| HYG/R | GAACCCGCGGTCGGCATCTACTCTAT |
| YG/F | GATGTAGGAGGGCGTGGATATGTCCT |
| HY/R | GAACCCGCGGTCGGCATCTACTCTAT |
| G1 | CCACCCACCAGCACACTA |
| G2 | CGGAAGCCTCATCAACAA |
| F3 | ATCTGTCCTTCTGTGTCCGA |
| R3 | CAAATGATGGTGTTCCCGA |
| H2F  H2R | TTCCTCCCTTTATTTCAGATTCAA  ATGTTGGCGACCTCGTATTGG |
| H1F | GTCGATGCGACGCAATCGT |
| H1R | GCTGATCTGACCAGTTGC |
| cpF | CTATAGGGCGAATTGGGTACCCTCACAGTCACATCCACGAG |
| cpR | GCAGGCATGCAAGCTTATCGATGTCGTAGATGTCTTGATCC |
| neiF | CACCTGGATACGTCGGTC |
| GFPR | GATGCCCTTCAGCTCGATGCGGTTCA |
| RTF | CAATACGGTCATTCTCAGCT |
| RTR | GTGCTCGCTCAACCACTT |
| TEF1a-RTF | TCACCACTGAAGTCAAGTCC |
| TEF1a-RTR | ACCAGCGACGTTACCACGTC |
| FpActin-RTF | ACCGTGAGAAGATGAC |
| FpActin-RTR | CGAAACCCTCGTAAATG |
| FPSE_02736-F | ATGACACTCGACACTACC |
| FPSE_02736-R | AACAAAGAGGCTGCTTCC |
| FPSE_01067-F | GACCTGTCAAGAACTCGAAG |
| FPSE_01067-R | CTGAGTTCGGATACAGTTG |
| FPSE_04527-F | TTGCCGTCACTCAAGTCA |
| FPSE_04527-R | AGCCTGCCAAGATATGAAGG |
| FPSE_02622-F | CGTTTGGATTCCTTACGAC |
| FPSE_02622-R | CTACGATTGCTTCATTCTTC |
| FPSE_11746-F | TGGTGGATGTATTGCAAGC |
| FPSE_11746-R | CGGTGATAAGAAGTGTCAG |
| FPSE_01933-F | CCAAGGTATATGTCAAGC |
| FPSE_01933-R | GCAACTGTAAATGAAGCCTG |
| FPSE_11531-RTF | CACCTATTCACGCATACC |
| FPSE_11531-RTR | GTGGTGGCGGAGGAGGAG |
| FPSE_11893-RTF | TCCAGCAAGCTATAACGG |
| FPSE_11893-RTR | GTGTTGCGGAATAGAGCT |
| FPSE_00757-RTF | AGCCAACCCATGTCCTGC |
| FPSE_00757-RTR | CATATTGCCGAATTCATGAG |
| FPSE_02660-RTF | ATGCTTGACCACCCTTCCG |
| FPSE_02660-RTR | GCGAGTGCGAGACTACGAG |
| FPSE_11664-RTF | GACTTGCGATGCTTTCACT |
| FPSE_11664-RTR | ACCTCTGCTGTCTTGGGTC |
| FPSE_00184-RTF | ATCTGTGGATTGGTGGTCG |
| FPSE_00184-RTR | TTCAGGGTCCTCAGGGTAA |
| FPSE_04141-F | TATCATGATCGGTATGGG |
| FPSE_04141-R | TAAGATCACGACCAGCCA |
