# Supplementary material for: Heat Stress Tolerance Gene FpHsp104 Affects Conidiation and Pathogenicity of Fusarium pseudograminearum
Source: Front Microbiol. 2021 Jul 28;12:695535. doi: 10.3389/fmicb.2021.695535 (PMC8355993; doi:10.3389/fmicb.2021.695535)
Supplement: Supplementary file 2 [file Data_Sheet_1.docx]

Supplementary Material

**Supplementary Figure 1.** Transcriptional profiles of FpHsp104 gene. Transcript level fold of the FpHsp104 gene was calculated relative to the level at MY. The FpActin gene was used as a reference. Bars represent standard deviation from four qRT-PCR replicates. * *P* < 0.05, ** *P* < 0.01 (*t*-test).

**Supplementary Figure 2.** qRT-PCR analyses of *FpHsp104* in WT and *Δfphsp104* strains. Transcript level fold of *FpHsp104* gene was calculated relative to the level at WT. The *FpTEF1a* gene was used as a reference. Bars represent standard deviation based on four replicates. ** *P* < 0.01 (*t*-test).

**Supplementary Figure 3.** Growth of mycelia and conidia under high temperature. (**A**) Phenotypes of WT, Δfphsp104, and *Δfphsp104*-C strains cultured on MM at 25 °C for 1.5 d, 2.5 d (CK), 4.5 d (CK), exposed to 34 °C for 1 d (2.5 d-HS), and returned to the optimal temperature (25 °C) for 2d (4.5 d-HS). (**B**) Statistical analysis of colony diameter. Colony diameter is presented as the mean ± standard deviation of at least three independent experiments. ***P*<0.01, **P*<0.05 (*t*-test). (**C**) Hyphal morphology of WT, *Δfphsp104*, and *Δfphsp104*-C strains cultured on MM at 25 °C (CK), exposed to 34 °C for 1 d (HS), and returned to 25 °C for 2 d (RE). Bar = 20 μm. (**D**) Equal amount of conidia of WT, *Δfphsp104*, and *Δfphsp104*-C were exposed to 34 °C for 2 h or not, and then inoculated onto MM plates at 25 °C for 3 d.

**Supplementary Figure 4.** qRT-PCR analyses of nine conidiation-related genes in WT and *Δfphsp104* strains. Transcript level fold of the selected gene was calculated relative to the level at WT. The *FpActin* gene was used as a reference. Bars represent standard deviation based on four replicates. ** *P* < 0.01, * *P* < 0.05 (*t*-test). *FpFlbC*, FPSE_02736; *FpSteA*, FPSE_01067; *FpFluG*, FPSE_04527; *FpMedA*, FPSE_01933; *FpVosA*, FPSE_11893; *FpBrlA*, FPSE_00757; *FpWetA*, FPSE_02660; *FpAbaA*, FPSE_11664; *FpPkaC*, FPSE_00184.

**Supplementary Figure 5.** Conidia germination assays. (**A**) Microscopy images of conidia formation induced in sterile water, PDB and MM for 3-6 h. Bar = 20 μm. (**B**) Conidia germination rates of WT, *Δfphsp104*, and *Δfphsp104*-C strains cultured in sterile water, PDB and MM for 6 h, respectively. Bars represent SD of three independent experiments. ***P*<0.01, * *P*<0.05 (*t*-test).

**Supplementary Table 1.** Primers used in the study.
