# Supplementary figures and images for: Heat Stress Tolerance Gene FpHsp104 Affects Conidiation and Pathogenicity of Fusarium pseudograminearum
Source: Front Microbiol. 2021 Jul 28;12:695535. doi: 10.3389/fmicb.2021.695535 (PMC8355993; doi:10.3389/fmicb.2021.695535)

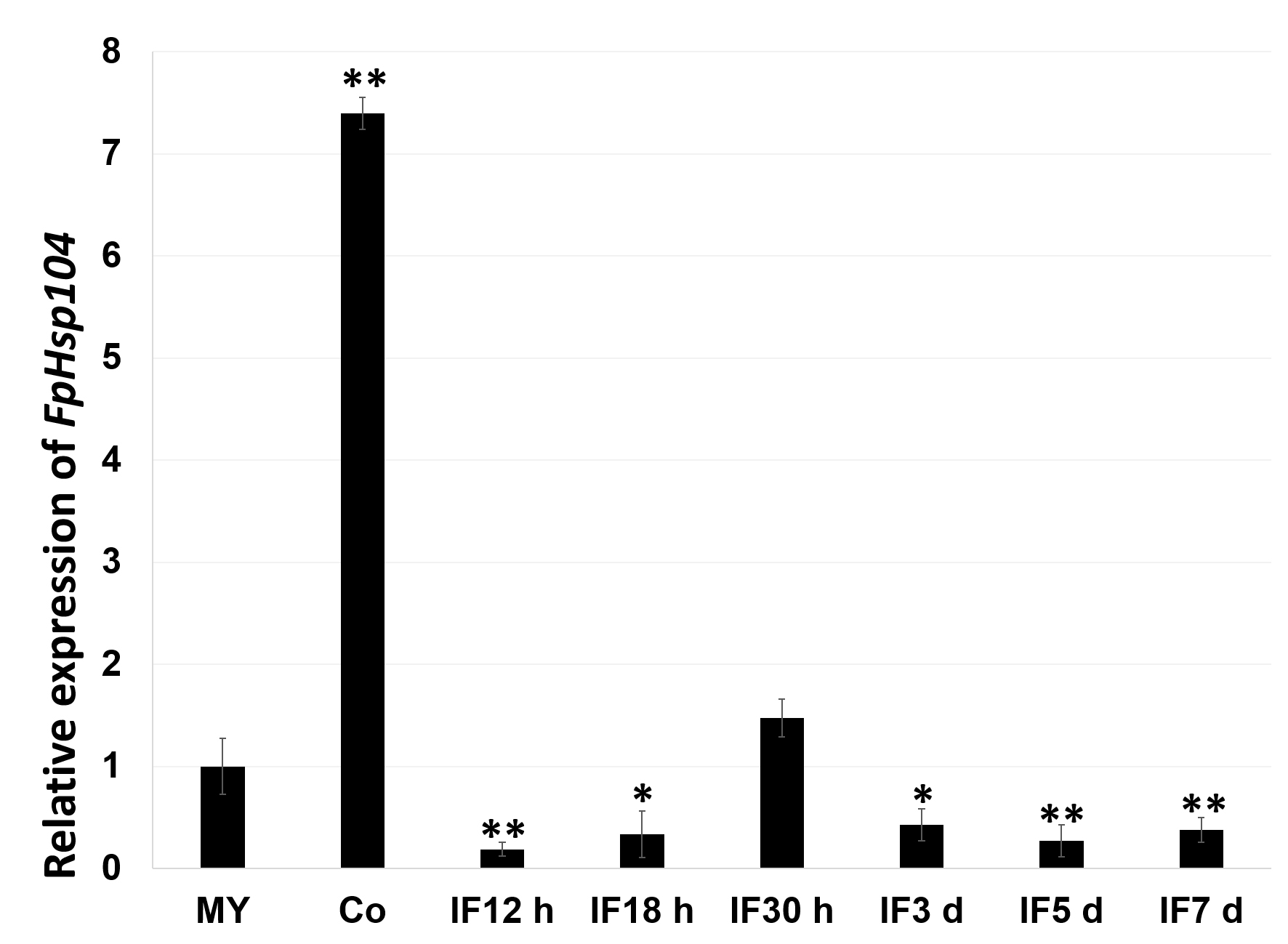

Supplement: Supplementary file 3 [file Image_1.JPEG]

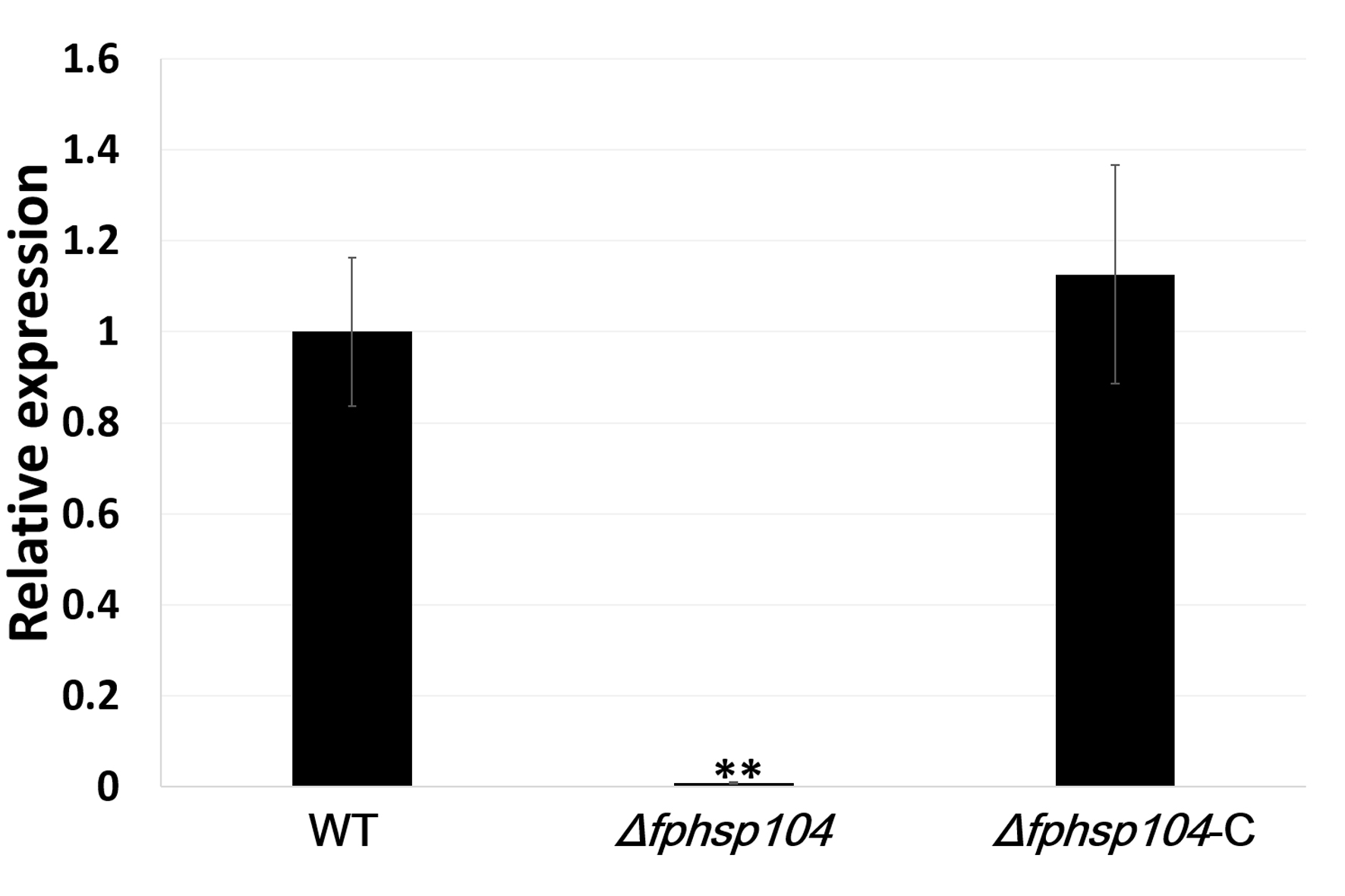

Supplement: Supplementary file 4 [file Image_2.JPEG]

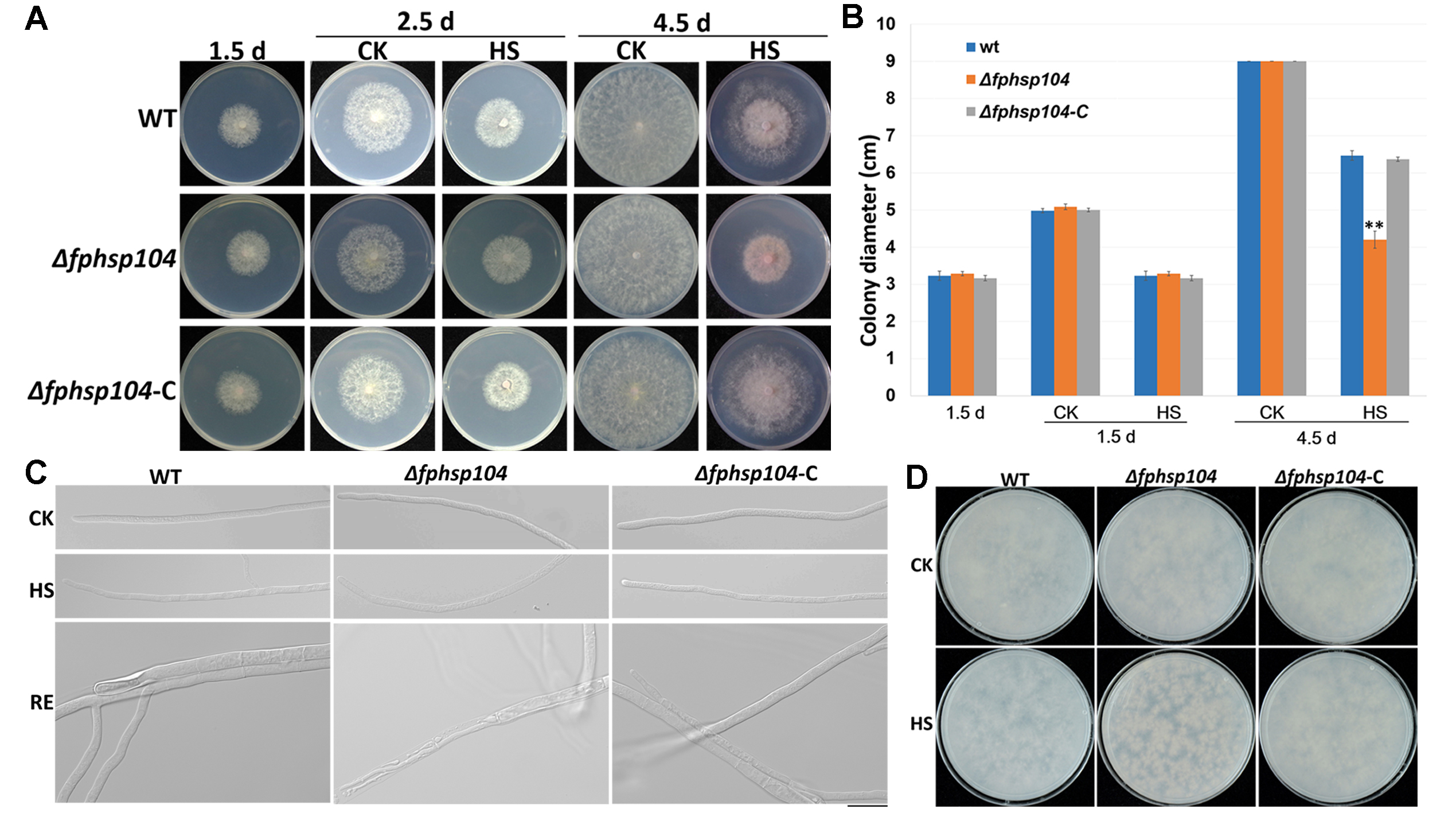

Supplement: Supplementary file 5 [file Image_3.JPEG]

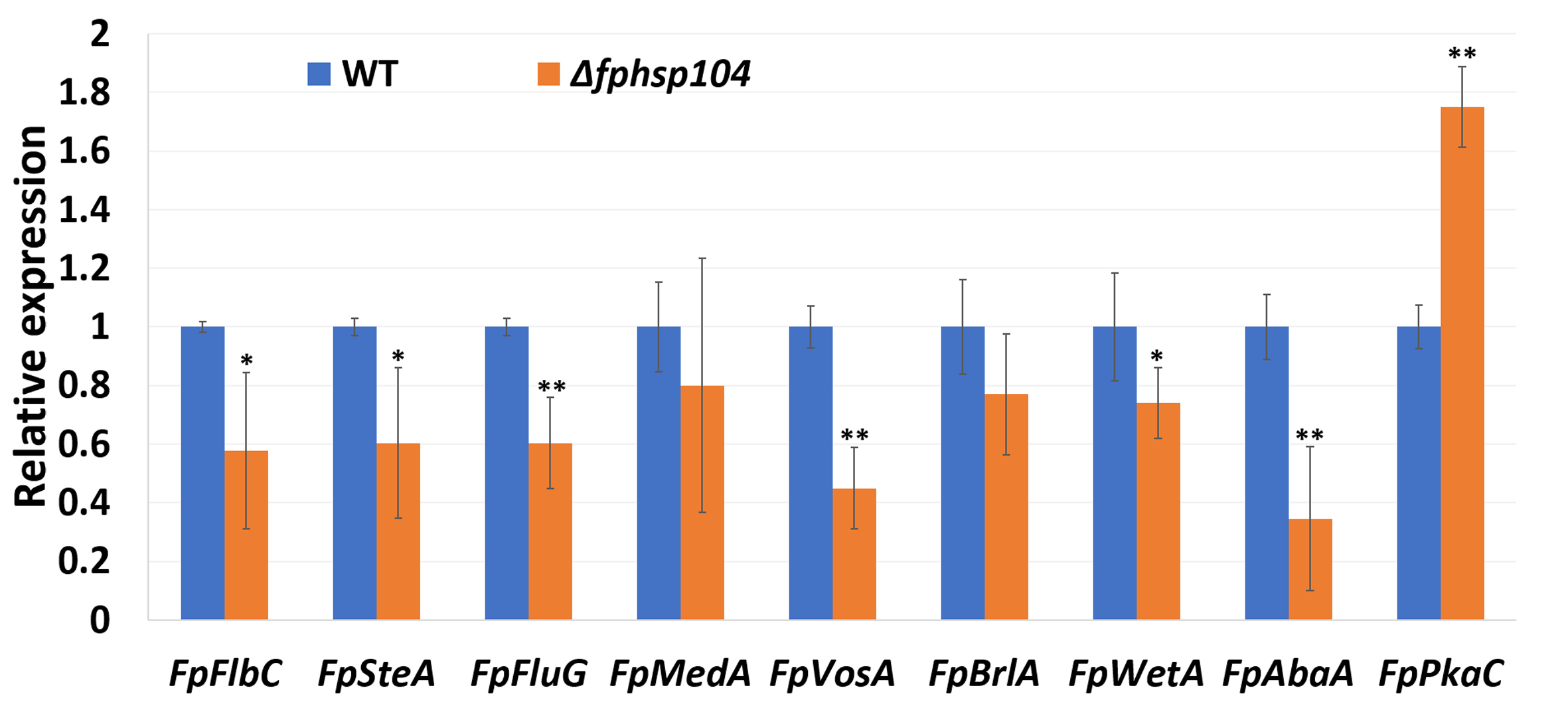

Supplement: Supplementary file 6 [file Image_4.JPEG]

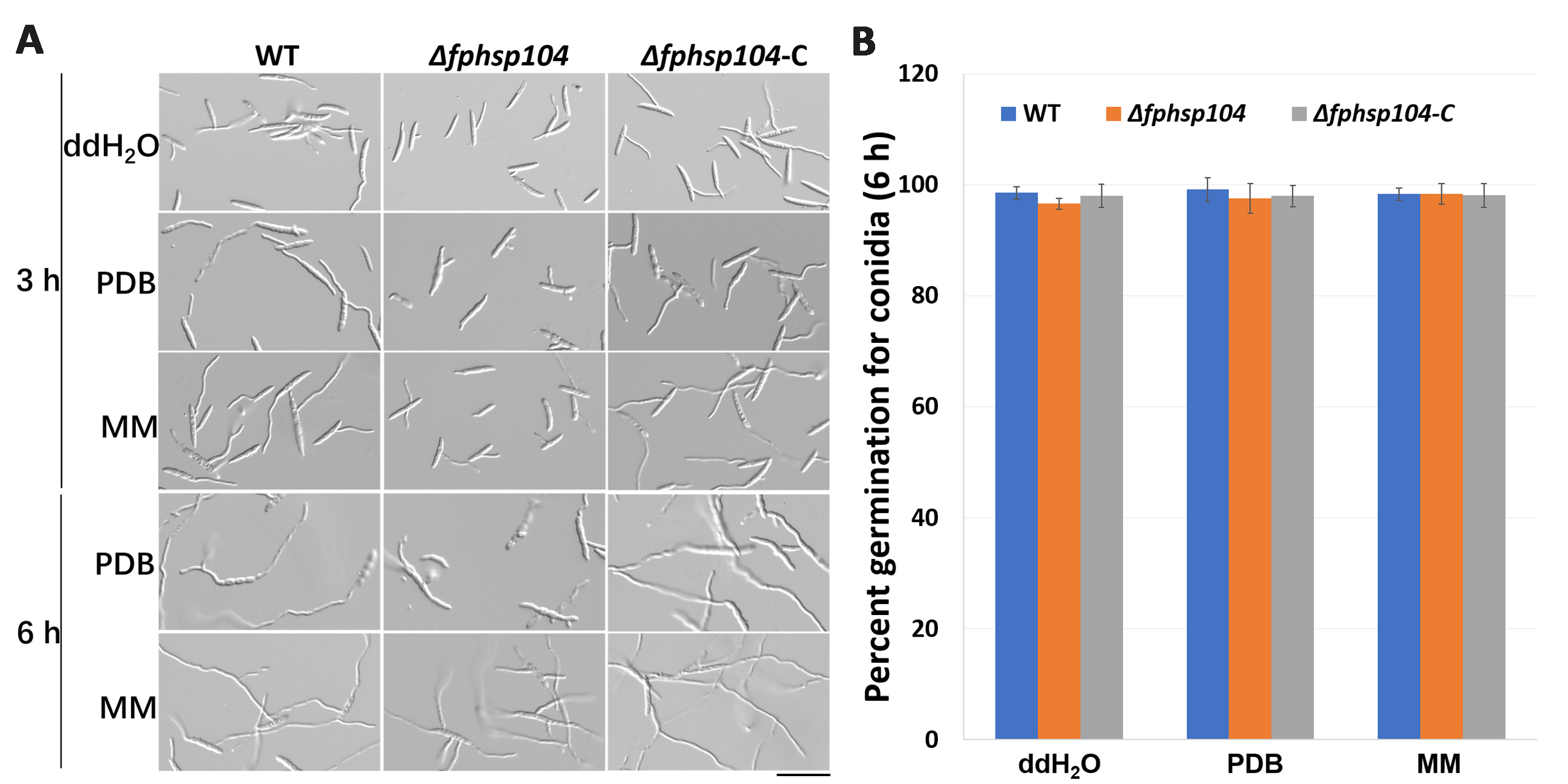

Supplement: Supplementary file 7 [file Image_5.JPEG]
